# Alkyl‐π Liquids as Condensed‐State Singlet Oxygen Photosensitizers

**DOI:** 10.1002/chem.202500739

**Published:** 2025-05-19

**Authors:** Ravindra Kumar Gupta, Takashi Nakanishi, Daniel T. Payne

**Affiliations:** ^1^ Research Center for Materials Nanoarchitectonics (MANA) National Institute for Materials Science (NIMS) Namiki 1‐1 Tsukuba Ibaraki 305‐0044 Japan; ^2^ International Center for Young Scientists (ICYS) National Institute for Materials Science (NIMS) Namiki 1‐1 Tsukuba Ibaraki 305‐0044 Japan; ^3^ School of Life, Health and Chemical Sciences The Open University Walton Hall Milton Keynes MK7 6AA UK

**Keywords:** alkyl‐π liquids, chromophores, functional materials, functional molecular liquids, singlet oxygen photosenstizers

## Abstract

Functional materials capable of generating singlet oxygen (^1^O_2_), a highly reactive but short‐lived species used to destroy organic materials, including chemical pollutants and biological entities, typically incorporate a chromophore that acts as a photosensitizer into a tertiary scaffold. Current functional materials that produce ^1^O_2_ include metal‐organic frameworks (MOFs), covalent‐organic frameworks (COFs), polymeric nanoparticles, modified glasses, and supramolecular assemblies. Whilst multi‐component functional materials have been widely reported, producing functional materials using a single small molecule in a condensed state has hardly been reported. Herein, we report the first use of functional molecular liquids (alkyl‐π liquids), non‐volatile single‐component condensed‐state fluidic materials, as photosensitizers for the generation of ^1^O_2_ at an alkyl‐π liquid‐water interface. We investigate the incorporation of various chromophores into alkyl‐π liquids that are suitable for ^1^O_2_ production and analyze the molecular structure required to produce efficient alkyl‐π liquid photosensitizers. The alkyl‐π liquids were studied, impregnated into porous membranes, and as thin films on quartz and Si wafers, the limitations of ^1^O_2_ production were investigated. A system was successfully fabricated that can generate ^1^O_2_ within an alkyl‐π liquid impregnated membrane and migrate across a membrane‐water interface to destroy small organic molecules, demonstrating the potential of these systems for water decontamination.

## Graphical abstract

Alkyl‐π liquids are non‐volatile single‐component condensed‐state functional materials that generate singlet oxygen (^1^O_2_) at an alkyl‐π liquid‐water interface. Chromophores can be incorporated into alkyl‐π liquids that produce ^1^O_2_ and membrane impregnated systems can be fabricated to generate ^1^O_2_ that migrates across a membrane‐water interface to destroy small organic molecules, demonstrating the potential for water decontamination.

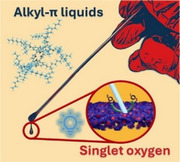

## 1 Introduction

Singlet oxygen (^1^O_2_) is a reactive oxygen species (ROS) formed by the excitation of diatomic oxygen from its triplet ground state to an excited singlet state.^[^ ^1^ ^]^ This is achieved using ^1^O_2_ photosensitizers, which, when irradiated with light, are excited into a triplet state that can transfer energy to ground‐state triplet oxygen, causing excitation to the singlet state.^[^ ^2^ ^]^ Several classes of chromophores can be used as ^1^O_2_ photosensitizers, including compounds containing extended π‐systems (pyrenes, anthracenes, etc.), porphyrins and related compounds, fullerenes, Rose Bengal analogues, phenalenone (PN), and tris(2,2‐bipyridine)ruthenium(II) salts.^[^ ^3^ ^] 1^O_2_ has several possible applications, including inorganic oxidative transformations,^[^ ^4^ ^]^ the destruction of environmental pollutants (especially for water purification),^[^ ^5^ ^]^ and most commonly in photodynamic therapy (PDT), including bacterial inactivation^[^ ^6^ ^]^ and cancer treatments.^[^ ^7^ ^]^ The effectiveness of ^1^O_2_ arises from its oxidative properties and high reactivity toward organic materials alongside its moderate lifetime (typically µs), which allows for the targeted generation of the species in situ.^[^ ^1^, ^2^ ^]^

^1^O_2_ photosensitizers are typically organic or organometallic chromophores with peripheral water‐solubilizing groups, as most ^1^O_2_ applications are carried out in an aqueous environment.^[^ ^7^, ^8^ ^]^ However, extended π‐systems, such as those in photosensitizers, are prone to aggregation in aqueous media, leading to chro mophore deactivation for ^1^O_2_ generation.^[^ ^9^ ^]^ This has led to the development of a variety of chromophore‐containing functional materials that can be dispersed in water for ^1^O_2_ production, including metal‐organic frameworks (MOFs),^[^ ^10^ ^]^ covalent‐organic frameworks (COFs),^[^ ^11^ ^]^ polymers,^[^ ^12^ ^]^ modified glasses,^[^ ^6^, ^13^ ^]^ and supramolecular assemblies.^[^ ^14^ ^]^ All examples require a multi‐component material architecture for suitable chromophore spacing to inhibit chromophore deactivation, which can be time‐consuming and tedious to produce. Recent studies into single‐component functional materials have had varied results; whilst nanomaterials can be produced readily,^[^ ^15^ ^]^ incorporating chromophores into single‐component macroscale materials for ^1^O_2_ generation is more challenging. Perylene‐based photosensitizers have been used to produce cm‐scale materials capable of generating ^1^O_2_. Langmuir–Blodgett lifting of a perylene bisimide (PBI) gave an LS film but with poor ^1^O_2_ generation once > 15 single‐molecule layers are deposited onto the substrate,^[^ ^16^ ^]^ and the drop‐casting of PBI‐dyads gave films capable of generating ^1^O_2_ but with no analysis of the thickness and how this affects efficiency.^[^ ^17^ ^]^

Alkyl‐π liquids are non‐volatile single‐component condensed‐state fluidic materials.^[^ ^18^ ^]^ The general molecular design of alkyl‐π liquids features a core unit (typically a polyaromatic conjugated π‐system) with numerous bulky yet flexible alkyl side chains, which contribute to the fluidic nature of the material at ambient temperature because of their relatively high entropy.^[^ ^19^ ^]^ These alkyl‐π liquids are usually non‐ionic and hydrophobic with very low glass transition temperatures (*T*_g_), meaning they can flow freely at room temperature and can be directly placed on and filled into various shapes and geometries, which is desirable for flexible devices.^[^ ^20^ ^]^ The π‐core is responsible for the functional nature of the liquid; for example, the inclusion of a chromophore imparts absorption‐emission properties on the alkyl‐π liquid, and chromophore modification allows for tuneable optical properties.^[^ ^21^ ^]^ The wrapping of the π‐core with alkyl side chains isolates the π‐core unit and provides additional physical properties, including high photochemical and thermal stability.^[^ ^21d^ ^]^ In addition, the π‐core isolation inhibits intermolecular π–π interactions, which maintains the inherent single molecular optical properties in the condensed state.^[^ ^21^, ^22^ ^]^ Alkyl‐π liquids have been utilized in various research fields, including luminescent inks,^[^ ^21^, ^23^ ^]^ liquid semiconductors,^[^ ^24^ ^]^ MOF like liquids with permanent porosity,^[^ ^25^ ^]^ organic light emitting diodes (OLEDs),^[^ ^26^ ^]^ and liquid electrets.^[^ ^20^, ^27^ ^]^

**Figure 1:**
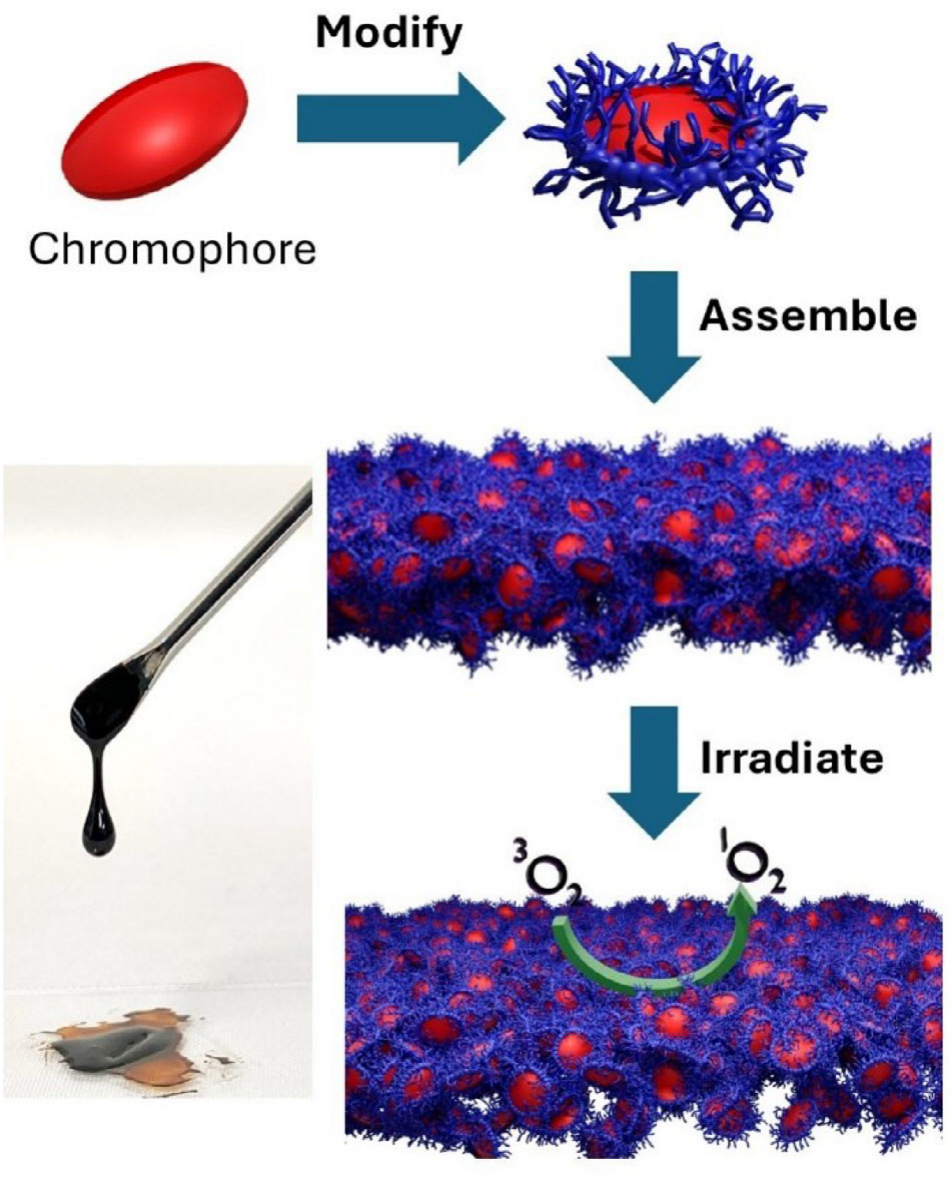
Schematic of the overall concept of this work and a representative image of an alkyl π‐liquid.

Whilst alkyl‐π liquids have been used as functional organic soft materials, including numerous optical applications, to date, they have not been used as ^1^O_2_‐generating functional materials, despite several examples having previously incorporated ^1^O_2_ photosensitizers capable of accessing an excited triplet state.^[^ ^23^, ^28^ ^]^ Alkyl‐π liquids have several beneficial properties for this application, including high photochemical and thermal stability, tunable excitation parameters, and limited π–π interactions between chromophore units. This study investigates the ability of alkyl‐π liquids to be used as condensed‐state functional materials containing ^1^O_2_ photosensitizers. Herein, a library of reported alkyl‐π liquids has been studied that contains three π‐core chromophores (anthracene, pyrene, and tetraphenylporphyrin) that have been modified with the same branched alkyl side chains (2‐hexyldecyl, C_6_C_10_) in varied substitution patterns, as this alkyl group has been shown to cause efficient π‐core isolation when assembled in alkyl‐π liquids (Figure 1).^[^ ^21^, ^27^, ^29^ ^]^ This study aims to (i) determine if the general molecular design for modified alkyl‐π liquids is capable of ^1^O_2_ generation, (ii) measure the efficiency of ^1^O_2_ generation by alkyl‐π liquids in a condensed state, and (iii) investigate the migration of ^1^O_2_ at the alkyl‐π liquid‐water interface.

## 2 Results and Discussion

**Figure 2:**
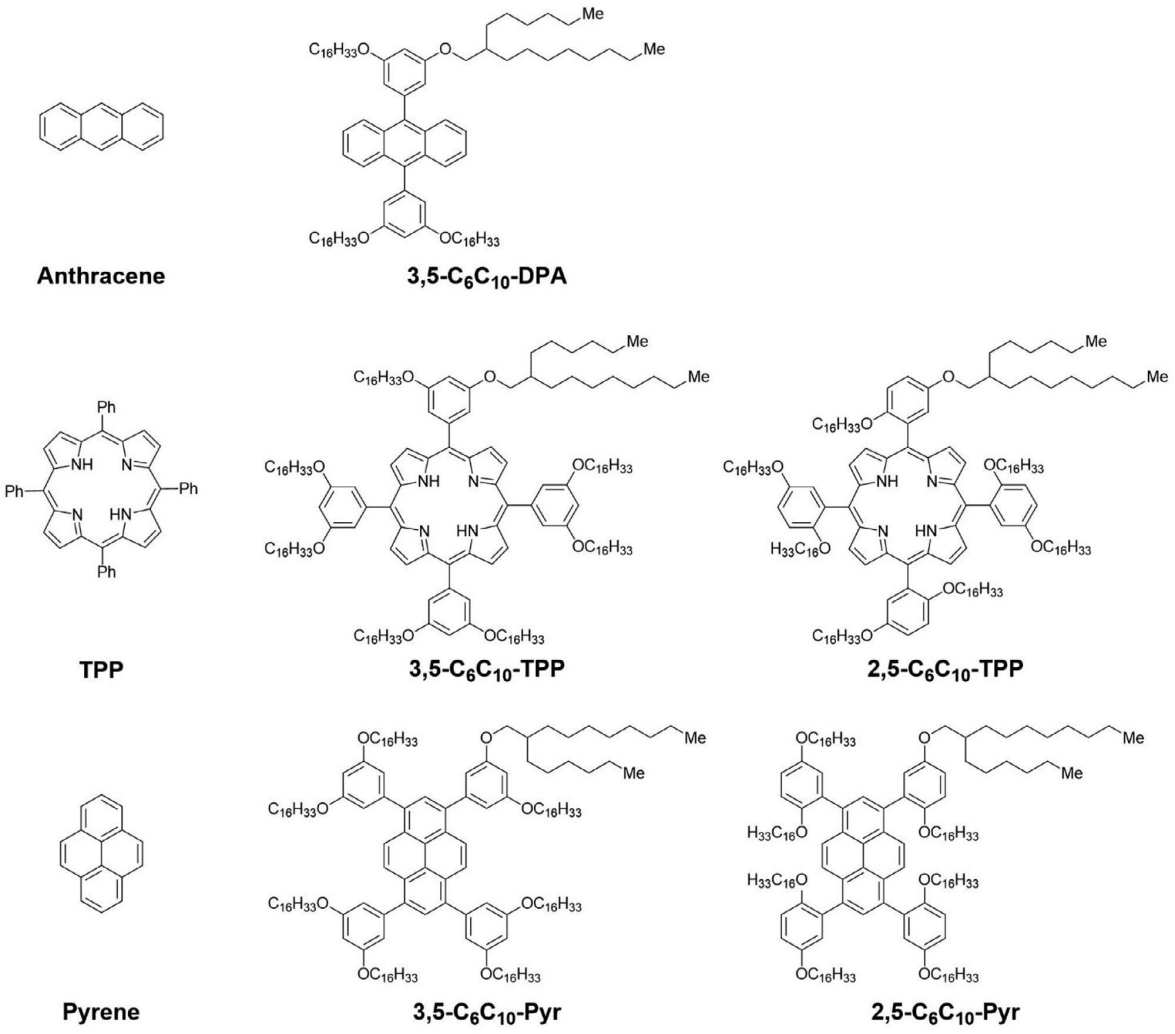
Chemical structures of the compounds studied in this work. Abbreviations of the compounds are given for ease of reference in the text.

The chemical structures of the alkyl‐π liquids and reference compounds used in this work are shown in Figure 2. A total of five previously reported alkyl‐π liquids were used in this study: **3,5‐C_6_C_10_‐DPA**,^[^ ^29^ ^]^ **3,5‐C_6_C_10_‐TPP**,^[^ ^27a^ ^]^ **2,5‐C_6_C_10_‐TPP**,^[^ ^27a^ ^]^ **3,5‐C_6_C_10_‐Pyr**,^[^ ^21c^ ^]^ and **2,5‐C_6_C_10_‐Pyr**.^[^ ^21c^ ^]^ Anthracene, tetraphenylporphyrin, and pyrene were used as chromophore cores as they have all been shown to generate ^1^O_2_ in organic solutions.^[^ ^3b^ ^]^ In addition, these chromophores are all common in alkyl‐π liquids, and information regarding their additional photophysical properties, related to the generation of ^1^O_2_, may be of importance to investigations into alternative applications. Porphyrin‐related photosensitizers are of significant interest, as the majority of currently used ^1^O_2_ photosensitizers in clinical settings include a porphyrin‐related chromophore.^[^ ^30^ ^]^ Whilst anthracene and pyrene‐based photosensitizers are less common due to their short wavelength absorption profiles (300–500 nm) and reported instability towards ^1^O_2_ in the solution state. Phenyl groups were included at the 9,10‐positions of anthracene and the 1,3,6,8‐positions of pyrene to allow for the addition of multiple alkyl side chains. Phenyl ether modifications in either 2,5‐ or 3,5‐ substitution patterns on the peripheral phenyl groups of the chromophores allowed for the installation of C_6_C_10_ alkyl chains, which induced the fluidic nature of the materials in the condensed state and facilitated the chromophore separation.

**Figure 3:**
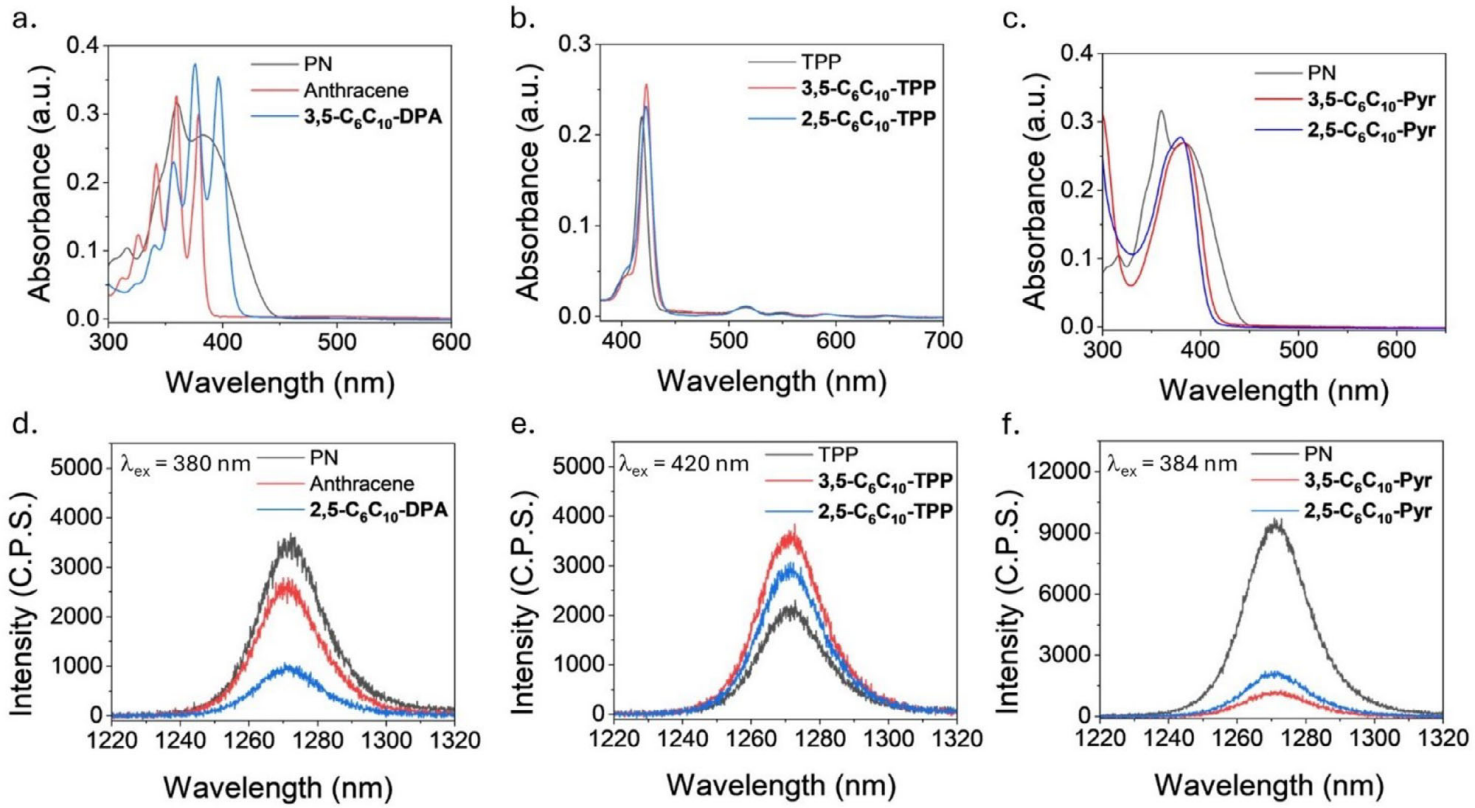
Electronic absorption and photoluminescence spectra for alkyl‐π liquids and reference compounds. a) UV‐vis spectra of chloroform solutions of anthracene, **3,5‐C_6_C_10_‐DPA**, and a reference (PN) having approximately equivalent absorbances at the wavelength of irradiation (380 nm). b) UV‐vis spectra of chloroform solutions of **3,5‐C_6_C_10_‐TPP**, **2,5‐C_6_C_10_‐TPP**, and a reference (TPP) having approximately equivalent absorbances at the wavelength of irradiation (420 nm). c) UV‐vis spectra of chloroform solutions of **3,5‐C_6_C_10_‐Pyr**, **2,5‐C_6_C_10_‐Pyr**, and a reference (PN) having approximately equivalent absorbances at the wavelength of irradiation (384 nm). d) ^1^O_2_ photoluminescence spectra of the chloroform solutions of anthracene, **3,5‐C_6_C_10_‐DPA**, and a reference (PN) under irradiation at 380 nm. e) ^1^O_2_ photoluminescence spectra of the chloroform solutions of **3,5‐C_6_C_10_‐TPP**, **2,5‐C_6_C_10_‐TPP**, and a reference (TPP) under irradiation at 420 nm. f) ^1^O_2_ photoluminescence spectra of the chloroform solutions of **3,5‐C_6_C_10_‐Pyr**, **2,5‐C_6_C_10_‐Pyr**, and a reference (PN) under irradiation at 384 nm.

To give an indication of the quantum yields of ^1^O_2_ generation (*Φ*_Δ_) for all compounds in solution, the electronic absorption intensity of the photosensitizer of study must be normalized (ca. 0.1–0.3 a.u.) with that of a reference material, with a similar absorption profile and a known *Φ*_Δ_, at an appropriately selected excitation wavelength in the same solvent.^[^ ^31^ ^]^ A reliable value for *Φ*_Δ_ was not available for anthracene in chloroform, and the need for absorption spectrum overlap meant that PN was required as an additional reference compound, which has a *Φ*_Δ_ of 0.98 in chloroform.^[^ ^3b^ ^]^ Anthracene and **3,5‐C_6_C_10_‐DPA** were normalized with PN to 0.27 a.u. at 380 nm (Figure 3a). **3,5‐C_6_C_10_‐TPP** and **2,5‐C_6_C_10_‐TPP** were normalized with TPP to 0.21 a.u. at 420 nm because TPP has a *Φ*_Δ_ of 0.55 in chloroform when irradiating the Soret band (Figure 3b).^[^ ^31^ ^]^ Both **3,5‐C_6_C_10_‐Pyr** and **2,5‐C_6_C_10_‐Pyr** were normalized with PN to 0.27 a.u. at 384 nm (Figure 3c), whereas pyrene was normalized with PN to 0.17 a.u. at 341 nm due to its blue‐shifted absorption profile compared to the alkyl‐π liquid derivatives (Figure S1).

*Relative phosphorescence intensity* values were determined by measuring the intensity of ^1^O_2_ photoluminescence (ca. 1260–1270 nm) relative to that of a reference compound from the normalized chloroform solutions of the compounds under irradiation at the wavelength of normalization. All alkyl‐π liquids generated ^1^O_2_ in solution (Figure 3d–f), and the results are summarized in Table 1. Variations in *relative phosphorescence intensity* were observed depending on molecular structure, where **3,5‐C_6_C_10_‐DPA** had a lower *relative phosphorescence intensity* than anthracene (0.28 vs. 0.74), and both **3,5‐C_6_C_10_‐Pyr** and **2,5‐C_6_C_10_‐Pyr** had a lower *relative phosphorescence intensity* than pyrene (0.12 and 0.22 vs. 0.59). Whereas **3,5‐C_6_C_10_‐TPP** and **2,5‐C_6_C_10_‐TPP** both had a considerably higher *relative phosphorescence intensity* compared to TPP (0.93 and 0.76 vs. 0.55), which is consistent with *Φ*_Δ_ trends reported for similar TPP derivatives.^[^ ^31^ ^]^ While the relative phosphorescence intensities compared to known photosensitizers may give an indication of the approximate *Φ*_Δ_ for the solubilized alkyl‐π liquids, the effect of the C_6_C_10_ alkyl chains on ^1^O_2_ is not known and may affect the accuracy of these measurements. Stability in solution of the alkyl‐π liquids compared to the commonly used reference compounds was assessed by continuous irradiation of normalized solutions of the compounds at the absorption maxima whilst monitoring the ^1^O_2_ emission signal intensity at 1270 nm (Figure S2). Anthracene, pyrene, and **3,5‐C_6_C_10_‐DPA** showed some instability over 20 minutes of continuous irradiation, most likely due to a reaction with ^1^O_2_ causing chromophore degradation and photodimerization in the case of anthracene (diphenylanthracene derivatives are less prone to dimerization when irradiated).^[^ ^32^ ^]^ **3,5‐C_6_C_10_‐TPP**, **2,5‐C_6_C_10_‐TPP**, **3,5‐C_6_C_10_‐Pyr**, and **2,5‐C_6_C_10_‐Pyr** all show good photostability in solution compared to PN and TPP, and the addition of the peripheral phenyl groups improved the stability of the pyrene alkyl‐π liquids compared to pristine pyrene. An upward trend in phosphorescence intensity is observed over time for the three porphyrin‐based compounds, which was shown to be repeatable over two irradiation cycles (Figure S2b), which could be related to an increase in sample temperature during irradiation, as this was not controlled throughout the experiment.

**Table 1:** Summary of electronic absorption properties and ^1^O_2_ quantum yields for alkyl‐π liquids and reference compounds in chloroform.

Compound	UV‐vis *λ*_max_/nm	Reference [Φ_Δ_^ref^]	*λ*_ex_/nm	Relative phosphorescence intensity
Anthracene	378, 359, 342	PN (0.98)	380	0.74
**3,5‐C_6_C_10_‐DPA**	396, 377, 357	PN (0.98)	380	0.28
**3,5‐C_6_C_10_‐TPP**	423	TPP (0.55)	420	0.93
**2,5‐C_6_C_10_‐TPP**	422	TPP (0.55)	420	0.76
Pyrene	338, 322	PN (0.98)	341	0.59
**3,5‐C_6_C_10_‐Pyr**	383	PN (0.98)	384	0.12
**2,5‐C_6_C_10_‐Pyr**	379	PN (0.98)	384	0.22

**Figure 4:**
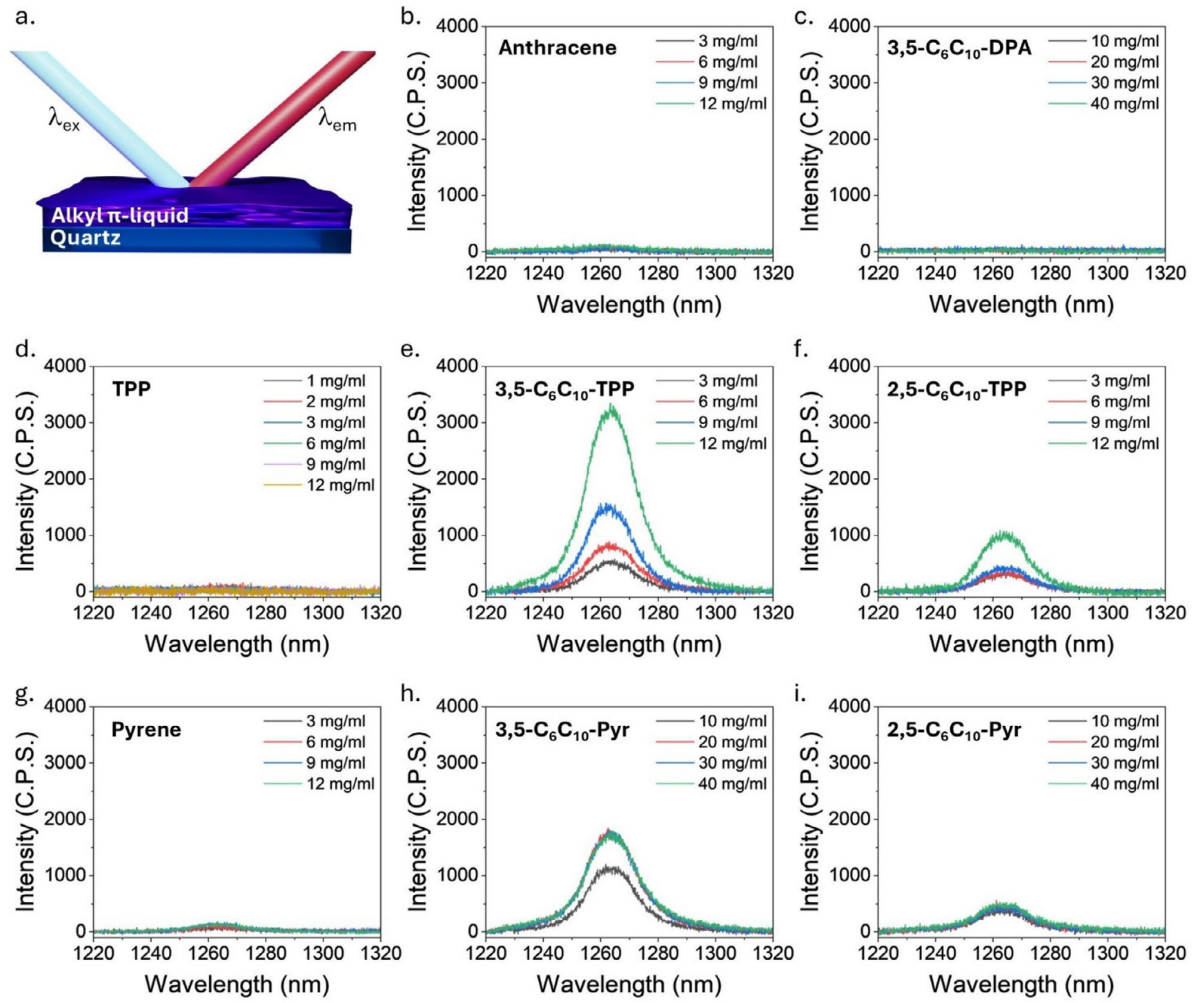
Photoluminescence spectra for alkyl‐π liquids and reference compounds as thin films on quartz. a) Schematic of the experimental setup. (b–i) ^1^O_2_ photoluminescence spectra of thin films of anthracene b), **3,5‐C_6_C_10_‐DPA** c), TPP d), **3,5‐C_6_C_10_‐TPP** e), **2,5‐C_6_C_10_‐TPP** f), pyrene g), **3,5‐C_6_C_10_‐Pyr** h), and **2,5‐C_6_C_10_‐Pyr** i) on quartz.

Whilst it was established that all alkyl‐π liquids and reference compounds could generate ^1^O_2_ in solution, to determine if they could generate ^1^O_2_ in a condensed state, thin films were prepared by spin coating alkyl‐π liquid solutions in chloroform at various concentrations onto quartz plates. The electronic absorption spectra for all samples showed that with increasing compound concentration in the spin coating solution, there was an increasing amount of compound present per unit area on the quartz plate based on the increasing absorbance intensity (Figure S3). Phosphorescence emission spectra for each thin film were recorded to determine if ^1^O_2_ was being generated upon irradiation (Figure 4a) and relative generation efficiencies can be inferred by comparison of absorbance values versus ^1^O_2_ emission intensities, but *Φ*_Δ_ cannot be calculated as there isn't a reference compound with a known quantum yield in a condensed‐state. Reference compounds anthracene (Figure 4b), TPP (Figure 4d), and pyrene (Figure 4g) were unable to generate ^1^O_2_ in a condensed state, likely due to the proximity of chromophores causing deactivation. **3,5‐C_6_C_10_‐DPA** also appeared to be unable to generate ^1^O_2_ when condensed into a thin film (Figure 4c); however, based on stability measurements (Figure S4), it appears that ^1^O_2_ was initially generated but quickly deactivated the film within 10 seconds, most likely due to endoperoxide formation.^[^ ^32^ ^]^ Both **3,5‐C_6_C_10_‐TPP** (Figure 4e) and **2,5‐C_6_C_10_‐TPP** (Figure 4f) generate ^1^O_2_ and showed good stability for > 20 minutes under continuous irradiation. Both showed near linear relationships between the Soret band absorbance value and ^1^O_2_ phosphorescence intensity, but **3,5‐C_6_C_10_‐TPP** has a greater phosphorescence intensity as an alkyl‐π liquid thin film, compared to thin films from **2,5‐C_6_C_10_‐TPP** with similar absorbance values, which may indicate more ^1^O_2_ is being generated by **3,5‐C_6_C_10_‐TPP** (Figure S5). Finally, **3,5‐C_6_C_10_‐Pyr** (Figure 4h) and **2,5‐C_6_C_10_‐Pyr** (Figure 4i) also generate ^1^O_2_ and showed good stability for > 20 minutes under continuous irradiation. However, the intensity of ^1^O_2_ phosphorescence does not correlate with the absorbance intensity of the alkyl‐pyrene liquid thin films (Figure S5).

**Figure 5:**
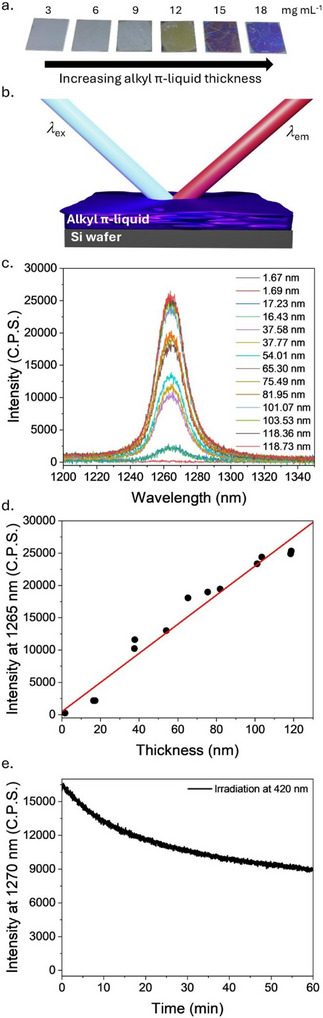
Analysis of **3,5‐C_6_C_10_‐TPP** films on Si wafer. (a) A series of samples prepared by spin‐coating with increasing concentration solution of **3,5‐C_6_C_10_‐TPP** in chloroform on Si wafer. (b) Schematic of the experimental setup for phosphorescence spectroscopy. (c) ^1^O_2_ photoluminescence spectra of thin films of **3,5‐C_6_C_10_‐TPP** on Si wafer under irradiation at 420 nm. (d) Correlation plot between film thickness and ^1^O_2_ phosphorescence intensity with a linear fit line. (e) Photostability of a thin film (12 mg mL^−1^) of **3,5‐C_6_C_10_‐TPP** by monitoring ^1^O_2_ photoluminescence intensity at 1270 nm during continuous irradiation at 420 nm.

The absorbance values for thin films on quartz indicate increasing film thickness as the concentration of the spin coating solution is increased, but it cannot be used to determine the absolute thickness value. Ellipsometry can be used to determine the thickness of a thin film, but the substrate must be changed from quartz to a Si wafer, as an opaque reflective surface is required to carry out accurate ellipsometry measurements.^[^ ^33^ ^]^ A range of thin films of varying thickness were prepared on a Si wafer (Figure 5a) and analyzed by ellipsometry to give an average thickness for the films (Table 2), which was determined by taking the average of a series of measurements across the films (Table S1). A linear relationship is obtained between the concentration of the spin coating solution and the film thickness (Figure S6) with average values ranging from approximately 16 to 120 nm. Attempts were made to obtain quantitative absorption spectra using an integrated sphere for the thin films on Si wafers (Figure S7), but the reflective nature of the substrate hindered the measurements, and no clear correlation could be found between absorbance and film thickness. Phosphorescence emission spectra for each thin film were recorded to determine if ^1^O_2_ was being generated upon irradiation (Figure 5c), and an increase in phosphorescence intensity is observed as the film thickness increases (Figure 5d and Table 2). A previous study using perylene‐based photosensitisers had observed that the limiting factor in thin film ^1^O_2_ generation produced by the Langmuir–Schaefer technique was aggregation of chromophores as film thickness was increased beyond 15 monomer layers.^[^ ^16^ ^]^ However, using alkyl‐π liquids appears to overcome this limitation, likely due to the alkyl side chains' ability to effectively isolate chromophores, as the results in Figure 5d indicate that thin film thickness trends linearly with the generation of ^1^O_2_. It appears that for alkyl‐π liquids, the limiting factors are most likely the amount of light per unit area being delivered to the alkyl‐π liquid surface, the adequate penetration depth of the light through the alkyl‐π liquid, and the diffusion of O_2_ through the alkyl‐π liquid. Finally, the photostability of an alkyl‐π liquid thin film was investigated by monitoring the ^1^O_2_ phosphorescence intensity during continuous irradiation at 420 nm of a sample produced through spin coating a 12 mg mL^−1^ solution of **3,5‐C_6_C_10_‐TPP** onto a Si wafer (Figure 5e) with an approximately 38% signal decrease observed over 60 minutes.

**Table 2:** Summary of **3,5‐C_6_C_1o_‐TPP** film thickness measured by ellipsometry and ^1^O_2_ phosphorescence signal intensities.

	Average thickness/nm		Intensity @ 1265 nm/C.P.S.	
3,5‐C_6_C_1o_‐TPP mass in CHCl_3_	Sample 1	Sample 2	Sample 1	Sample 2
0 mg mL^−1^	1.67 ± 0.01	1.69 ± 0.01	193	152
3 mg mL^−1^	17.23 ± 0.12	16.43 ± 0.72	2140	2111
6 mg mL^−1^	37.58 ± 0.21	37.77 ± 0.31	10,081	11,380
9 mg mL^−1^	54.01 ± 7.77	65.3 ± 1.19	13,062	17,900
12 mg mL^−1^	75.49 ± 3.73	81.95 ± 0.69	18,701	19,299
15 mg mL^−1^	101.07 ± 2.65	103.53 ± 3.50	23,256	24,195
18 mg mL^−1^	118.36 ± 1.86	118.73 ± 1.24	24,719	25,193

**Figure 6:**
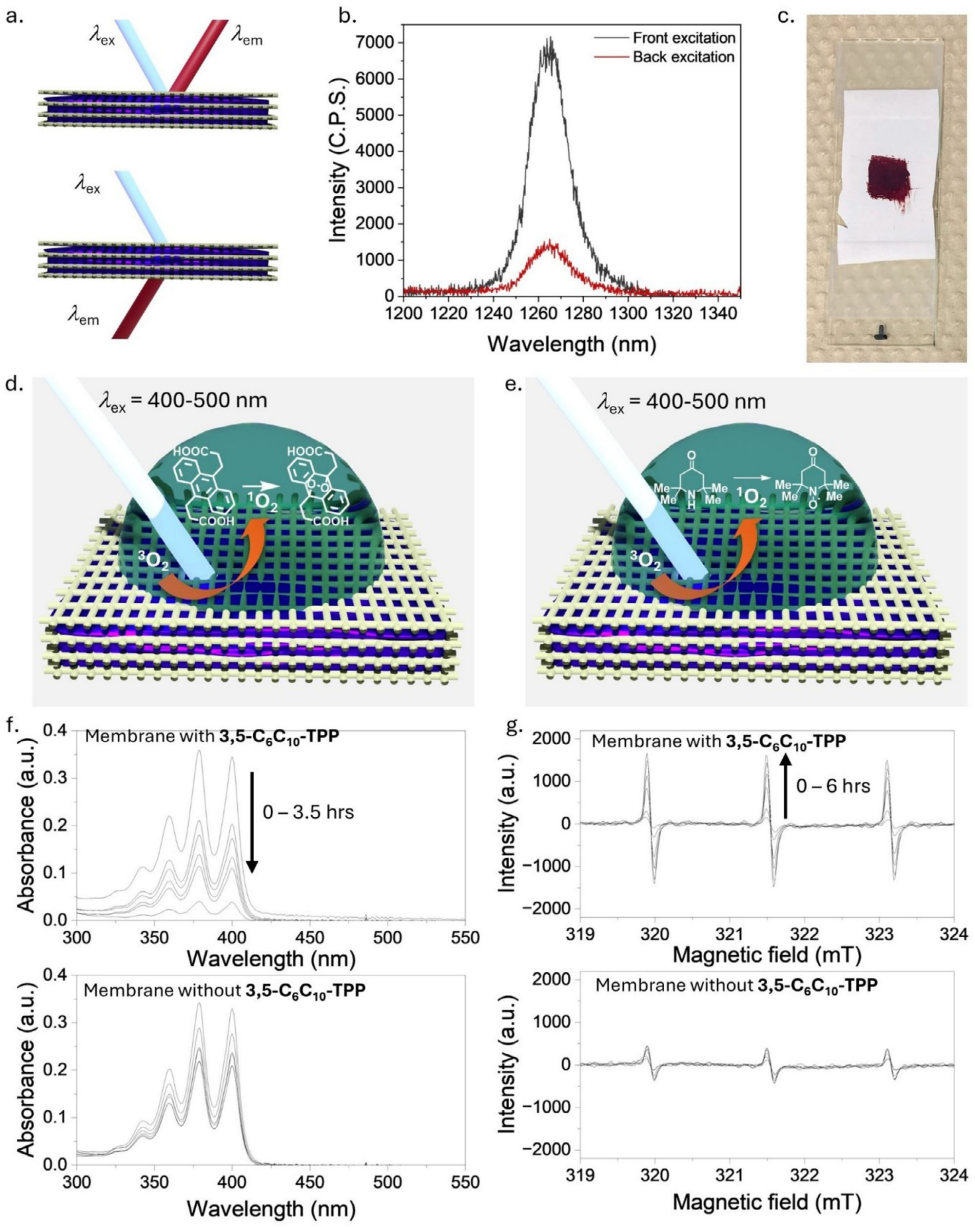
Analysis of **3,5‐C_6_C_10_‐TPP** permeated into a membrane. (a) Schematic of ^1^O_2_ phosphorescence spectroscopy measurements in either a front‐face (top) or back‐face (bottom) excitation‐emission arrangement. (b) ^1^O_2_ photoluminescence spectra of **3,5‐C_6_C_10_‐TPP** in membranes under irradiation at 420 nm. (c) Photos of a alkyl π‐liquid impregnated membrane used for the detection of ^1^O_2_ at the membrane‐water interface. (d) Schematic of the experimental setup for ^1^O_2_ trapping studies using an anthracene probe. (e) Schematic of the experimental setup for ^1^O_2_ trapping studies using an amine probe. (f) UV‐Vis ^1^O_2_ detection measurements using a basic solution of 3,3′‐(anthracene‐9,10‐diyl)dipropionic acid on membranes in the presence (top) and absence of **3,5‐C_6_C_10_‐TPP**. (g) EPR ^1^O_2_ detection measurements using a solution of TEMPD (2,2,6,6‐tetramethylpiperidone) on membranes in the presence (top) and absence of **3,5‐C_6_C_10_‐TPP**.

Most applications for ^1^O_2_ require an aqueous environment, but the alkyl‐π liquids in this study require alkyl side chains to induce fluidity, which renders the alkyl‐π liquids hydrophobic. While a water‐alkyl‐π liquid interface could be established by placing water droplets in contact with the alkyl‐π liquid on a quartz plate, over time, the alkyl‐π liquid migrates and spreads across the outer surface of the water droplet, which is not ideal for the application of these materials. Previous studies have utilized the mechanoelectrical properties of alkyl‐π liquids in wearable devices by permeating the alkyl‐π liquid into a flexible polyurethane membrane.^[^ ^27a^ ^]^ The same approach was used for ^1^O_2_ generating functional materials, and as the membrane is hydrophobic, the alkyl‐π liquid impregnates the pores but is not expelled when the membrane is placed in contact with water. The membrane was impregnated with alkyl‐π liquid by pasting it on the surface of the membrane, followed by standing at 50°C for 2 hours. Alternatively, the alkyl‐π liquid can be dissolved in chloroform and the membrane submerged, followed by slow evaporation. The generation of ^1^O_2_ by alkyl‐π liquid impregnated membranes was confirmed by ^1^O_2_ phosphorescence spectroscopy in either a front‐face or back‐face excitation‐emission arrangement (Figure 6a), with both arrangements giving an observable ^1^O_2_ phosphorescence signal (Figure 6b).

Whilst ^1^O_2_ could be detected at the membrane‐air interface, it is essential to ascertain whether this could be achieved at a membrane‐water interface (Figure 6c). Due to the short lifetime of ^1^O_2_, it is important to determine if it can be initially generated by the hydrophobic alkyl‐π liquids impregnated into the membrane and then diffuse into the water to carry out a function, such as the destruction of organic materials dissolved in the water. This analysis is not achievable using phosphorescence spectroscopy, as the spectrometer beam resolution would not be able to selectively excite and differentiate between ^1^O_2_ generated within the alkyl‐π liquid‐membrane at the membrane–air interface or at the membrane–water interface. Therefore, ^1^O_2_ trapping studies were carried out using either a water‐soluble anthracene (Figure 6d) or amine (Figure 6e) ^1^O_2_ trap monitored by either UV‐vis or EPR spectroscopy, respectively.^[^ ^15^, ^34^ ^]^ The cycloaddition of ^1^O_2_ to 3,3′‐(anthracene‐9,10‐diyl)dipropionic acid can be observed through the reduction of the anthracene UV‐vis response at 350–400 nm, which occurs faster in the presence of **3,5‐C_6_C_10_‐TPP** (Figure 6f and Figure S8b,c), however, the irradiation source led to some background photodegradation of the anthracene‐based probe due to photobleaching and possible dimerization. Therefore, the radical spin trap TEMPD (2,2,6,6‐tetramethylpiperidone) was used as an alternative approach to monitor the production of ^1^O_2_ through the formation of a stable radical. It can be monitored through the characteristic EPR response at 319–324 mT, which also occurs faster in the presence of the **3,5‐C_6_C_10_‐TPP**‐impregnated membrane (Figure 6g and Figure S8d). Fluctuations in the background signal for TEMPD in Figure 6g are due to small changes in concentration during the removal of the droplets from the membrane surface, as previous studies have shown light irradiation to have no measurable effect on TEMPD under continuous irradiation.^[^ ^15a^ ^]^ Both of these results confirm that ^1^O_2_ can be generated by an alkyl‐π liquid impregnated into a porous membrane and transferred across an interface with water to perform a function, including the destruction of organic materials, which could be beneficial for producing functional materials for water decontamination.

## 3 Conclusion

In conclusion, we have demonstrated that alkyl‐π liquids can be used as functional condensed‐state materials to produce ^1^O_2_. A range of alkyl‐π liquids with identical branched alkyl side chains (2‐hexyldecyl, C_6_C_10_) and varied chromophores known to be ^1^O_2_ photosensitizers (pyrene, anthracene, and tetraphenylporphyrin) were studied for their ability to generate ^1^O_2_ in chloroform solutions, with all compounds generating reasonable amounts compared to reference compounds. Alkyl‐π liquids containing porphyrin cores (**3,5‐C_6_C_10_‐TPP** and 2,5‐C_6_C_10_‐TPP) and pyrene cores (**3,5‐C_6_C_10_‐Pyr** and 2,5‐C_6_C_10_‐Pyr) were also able to generate ^1^O_2_ as solvent‐free thin films on quartz plates and Si wafers. Comparative analysis between the alkyl‐π liquids showed that **3,5‐C_6_C_10_‐TPP** generated the most ^1^O_2_ and trended linearly with film thickness. Whereas anthracene‐based alkyl‐π liquids were not suitable for the generation of ^1^O_2_ in solvent‐free thin films, likely due to the rapid formation of endoperoxides leading to deactivation of the chromophore. Finally, alkyl‐π liquids impregnated into porous membranes were fabricated with **3,5‐C_6_C_10_‐TPP** for the generation of ^1^O_2_ in aqueous media, which was confirmed through both UV‐vis analysis using an anthracene probe and EPR spectroscopy using an amine‐based radical spin trap. The ability to fabricate systems, such as alkyl‐π liquid impregnated into porous membranes and alkyl‐π liquid thin films, could be useful to produce systems for the decontamination of water, due to the ability of ^1^O_2_ to destroy organic pollutants and biological contaminants. The transfer of ^1^O_2_ across the alkyl‐π liquid‐water and membrane‐water interface demonstrated in our studies is of fundamental importance towards these future applications.

## 4 Experimental

Reagents and dehydrated solvents (in septum‐sealed bottles) used for syntheses and spectroscopic measurements were obtained from Tokyo Kasei Chemical Co., Wako Chemical Co., or Aldrich Chemical Co. and were used without further purification. Optical absorption spectra were measured using a JASCO V‐570 UV/Vis/NIR spectrophotometer. ^1^O_2_ photoluminescence spectra were measured using an InGaAs NIR photodetector (R5509‐73, Hamamatsu Photonics, Japan) on a NanoLog Horiba Jovin Yvon spectrofluorometer with a 450‐W xenon lamp as an excitation source at room temperature. A right‐angle detection method and quartz cuvettes with four optical faces that were usable in the UV field were used for emission measurements. ESR spectra were measured using a JEOL JES‐FA200 spectrometer with data recorded and processed using the A‐System version 1.6.5 PCI J/X‐Band and FAManager version 1.2.9 V2 series. Film thickness of Si wafers was measured using a high‐speed spectroscopic Ellipsometer (M‐2000U; J. A. Woollam Co., Inc., Lincoln, NE). All alkyl‐π liquids used in this study have been previously reported: **3,5‐C_6_C_10_‐DPA**,^[^ ^29^ ^]^ **3,5‐C_6_C_10_‐TPP**,^[^ ^27a^ ^]^ 2,5‐C_6_C_10_‐TPP,^[^ ^27a^ ^]^ **3,5‐C_6_C_10_‐Pyr**,^[^ ^21c^ ^]^ and 2,5‐C_6_C_10_‐Pyr.^[^ ^21c^ ^]^ POREFLON PTFE membrane was used as a porous membrane for material fabrication.

Film preparation: Quartz substrates (2 cm × 2 cm) were UV‐ozone treated and rinsed with ethanol prior to use. Silicon wafer substrates were cut into a size of 2 cm × 2 cm and thoroughly rinsed with IPA prior to use. Samples were dissolved in chloroform at various concentrations (e.g., 3, 6, 9, 12, and 18 mg mL^−1^), and 50 µL was spin‐coated onto quartz plates and Si wafers at 2000 rpm for 60 s.

Thickness measurements: The thickness of the spin‐coated film on a Si wafer was assessed using a high‐speed spectroscopic Ellipsometer. Each sample was duplicated and measured at five different points across the Si wafer, and an average of all measurements was taken for the final thickness result. To ensure the measurement accuracy, a blank silicon substrate was also measured with a thickness of ∼1.67 nm.

^1^O_2_ relative phosphorescence intensity measurements: Emission spectra and ^1^O_2_ photoluminescence spectra were measured using an NIR photodetector (Hamamatsu Photonics, Japan) on a NanoLog Horiba Jovin Yvon spectrofluorometer with a 450 W xenon lamp as an excitation source at room temperature under ambient conditions (unless otherwise stated). To estimate ^1^O_2_ quantum yields, the solutions of compounds were absorbance normalized (ca. 0.10–0.30 a.u.) with a reference compound at the relevant excitation wavelength in chloroform. Relative phosphorescence intensity was determined by comparison of the average ^1^O_2_ photoluminescence maxima values of the reference (*I*_ref_) and compound being studied (*I*_sample_) between 1263 and 1267 nm:

$$\mathrm{Relative}\;\mathrm{phosphorescence}\;\mathrm{intensity}=\frac{I_{\mathrm{sample}}}{I_{\mathrm{ref}}}\times \Phi_{\Delta }^{\mathrm{ref}}$$

EPR ^1^O_2_ measurements: A 100 µL aliquot of the 2,2,6,6‐tetramethylpiperidone (TEMPD, 0.26 M) solution in deionized water was placed on the membrane either with or without **3,5‐C_6_C_10_‐TPP** and irradiated with blue LEDs (400–500 nm) for a period (0.0, 1.0, 2.0, 3.0, 4.0, or 6.0 h). For irradiation times over 1 hour, 50 µL per hour of deionized water was added to the droplet to account for evaporation. After irradiation, the droplet was lifted from the membrane using a Hamilton 100 µL syringe, the volume of the droplet was measured and diluted to a final volume of 100 µL, and an EPR spectrum at each time point was recorded in a Drummond 100 µL microcap capillary sealed at one end under ambient conditions.

UV‐Vis ^1^O_2_ measurements: Sodium hydroxide 1 M (20 µL, 0.02 mmol, 3 equiv.) was added to a solution of 3,3′‐(anthracene‐9,10‐diyl)dipropionic acid (DPA, 2.22 mg, 0.0068 mmol, 1 equiv.) in deionized water (2.2 mL). A 50 µL aliquot of the DPA solution was placed on the membrane either with or without **3,5‐C_6_C_10_‐TPP** and irradiated with blue LEDs (400–500 nm) for a period (0.0, 1.0, 1.5, 2.5, 3.0, or 3.5 h). For irradiation times over 1 hour, 50 µL per hour of deionized water was added to the droplet to account for evaporation. After irradiation, the droplet was lifted from the membrane using a Hamilton 100 µL syringe, the volume of the droplet was measured and diluted to a final volume of 0.5 mL, and an absorption spectrum at each time point was recorded in a 1 mm path length quartz cell.

## Author Contributions

Daniel T. Payne and Takashi Nakanishi established the concept of the study. Ravindra Kumar Gupta performed alkyl‐π liquid film fabrication, electronic absorption spectroscopic measurements, and film thickness analysis. Daniel T. Payne performed phosphorescence spectroscopic measurements, EPR experiments, spin‐trap studies, and determined singlet oxygen quantum yields of the compounds. All authors contributed to the writing and editing of the manuscript.

## Conflict of Interests

The authors declare no conflict of interest.

## Supporting information



Supporting information

## Data Availability

The data that support the findings of this study are available from the corresponding author upon reasonable request.
