# Supplementary material for: Alkyl‐π Liquids as Condensed‐State Singlet Oxygen Photosensitizers
Source: Chemistry. 2025 May 19;31(33):e202500739. doi: 10.1002/chem.202500739 (PMC12160971; doi:10.1002/chem.202500739)
Supplement: Supplementary file 1 — Supporting information [file CHEM-31-e202500739-s001.docx]

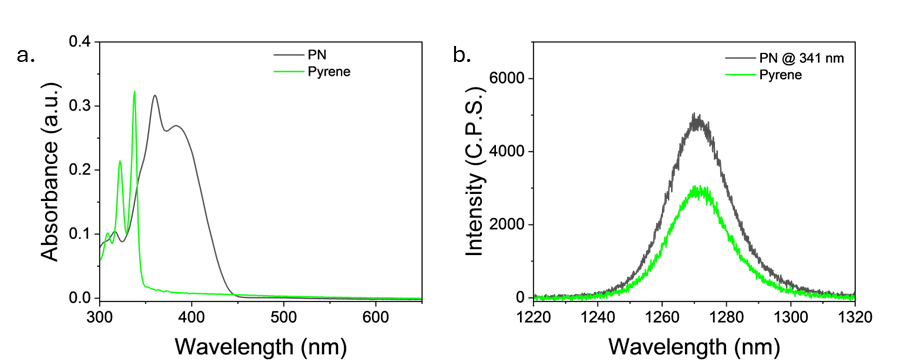


***Figure S1.*** *Electronic absorption and photoluminescence spectra for pyrene. (a) UV-vis spectra of chloroform solutions of pyrene and a reference (PN) having approximately equivalent absorbances at the wavelength of irradiation (341 nm). (b) ^1^O_2_ photoluminescence spectra of the chloroform solutions of pyrene and a reference (PN) under irradiation at 341 nm.*


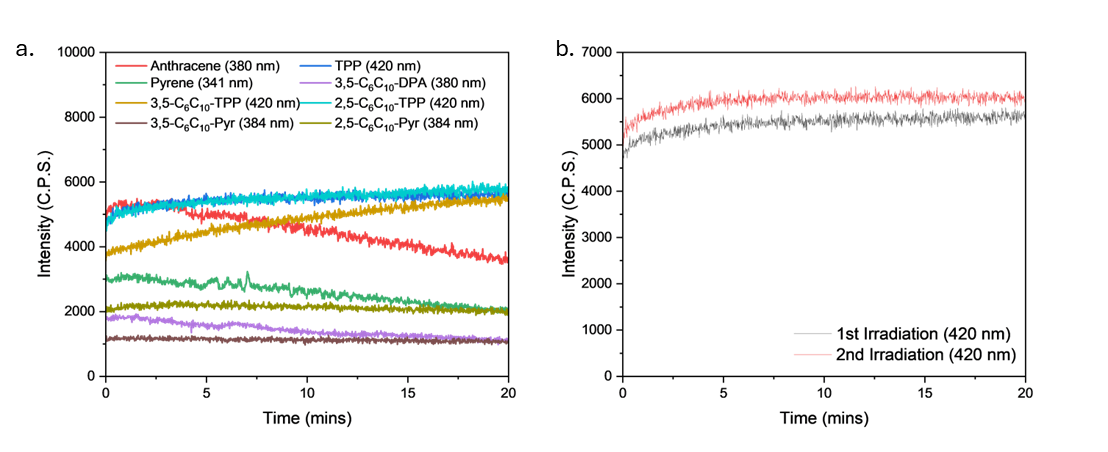


***Figure S2.*** *Stability of photosensitisers in solution determined by monitoring of ^1^O_2_ photoluminescence intensity at 1270 nm during continuous irradiation at the wavelength stated in the legend. a. Photostability of all compounds in chloroform. b. Photostability of TPP in chloroform after two sequential irradiation processes.*


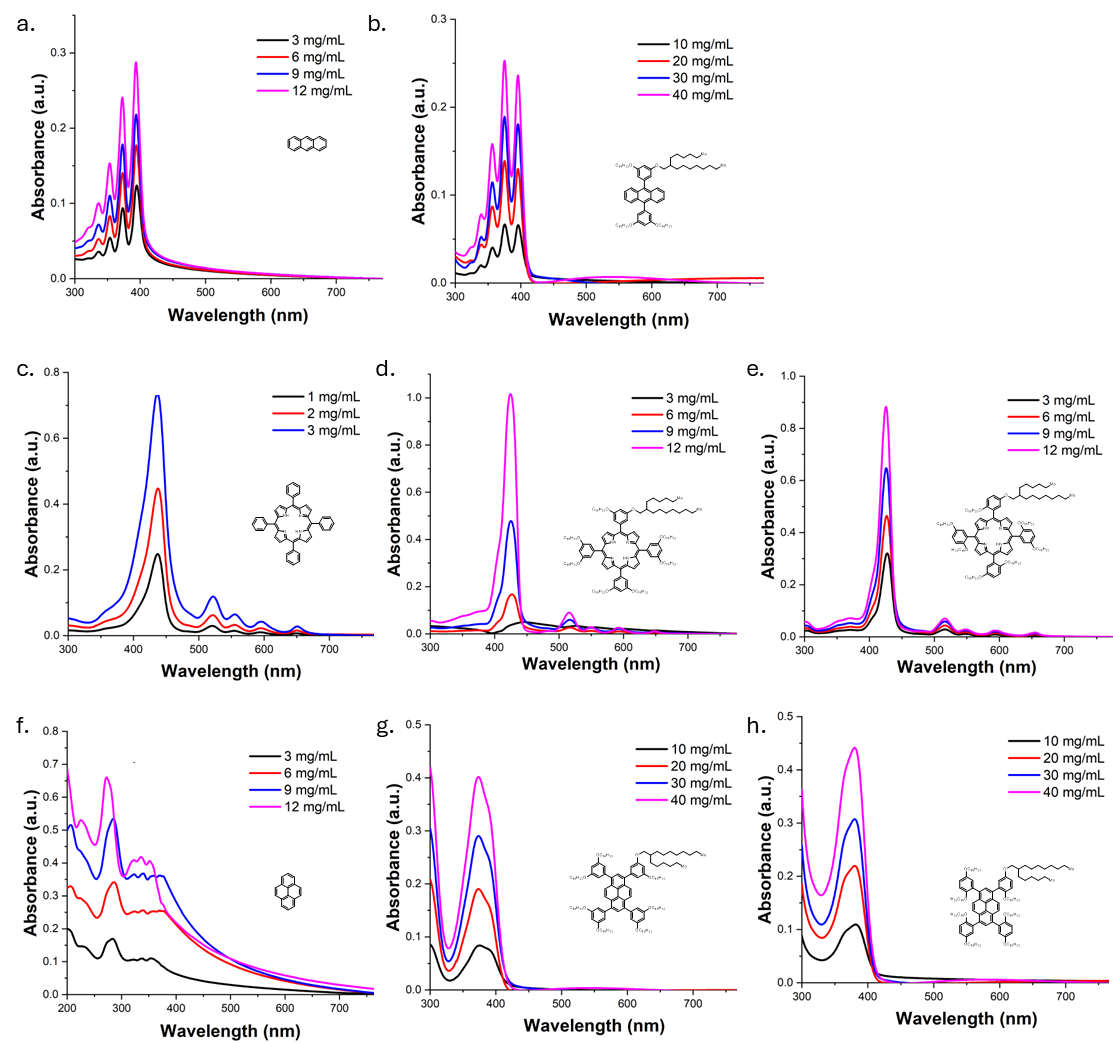


***Figure S3.*** *Electronic absorption spectra for alkyl-π liquids and reference compounds as thin films on quartz. (a) UV-vis spectra of thin films produced from spin coated 3 - 12 mg mL^-1^ chloroform solutions of anthracene on quartz. (b) UV-vis spectra of thin films produced from spin coated 10 - 40 mg mL^-1^ chloroform solutions of* ***3,5-C_6_C_10_-DPA*** *on quartz. (c) UV-vis spectra of thin films produced from spin coated 1 - 3 mg mL^-1^ chloroform solutions of TPP on quartz. (d) UV-vis spectra of thin films produced from spin coated 3 - 12 mg mL^-1^ chloroform solutions of* ***3,5-C_6_C_10_-TPP*** *on quartz. (e) UV-vis spectra of thin films produced from spin coated 3 - 12 mg mL^-1^ chloroform solutions of* ***2,5-C_6_C_10_-TPP*** *on quartz. (f) UV-vis spectra of thin films produced from spin coated 3 - 12 mg mL^-1^ chloroform solutions of pyrene on quartz. (g) UV-vis spectra of thin films produced from spin coated 10 - 40 mg mL^-1^ chloroform solutions of* ***3,5-C_6_C_10_-Pyr*** *on quartz. (e) UV-vis spectra of thin films produced from spin coated 10 - 40 mg mL^-1^ chloroform solutions of* ***2,5-C_6_C_10_-Pyr*** *on quartz.*


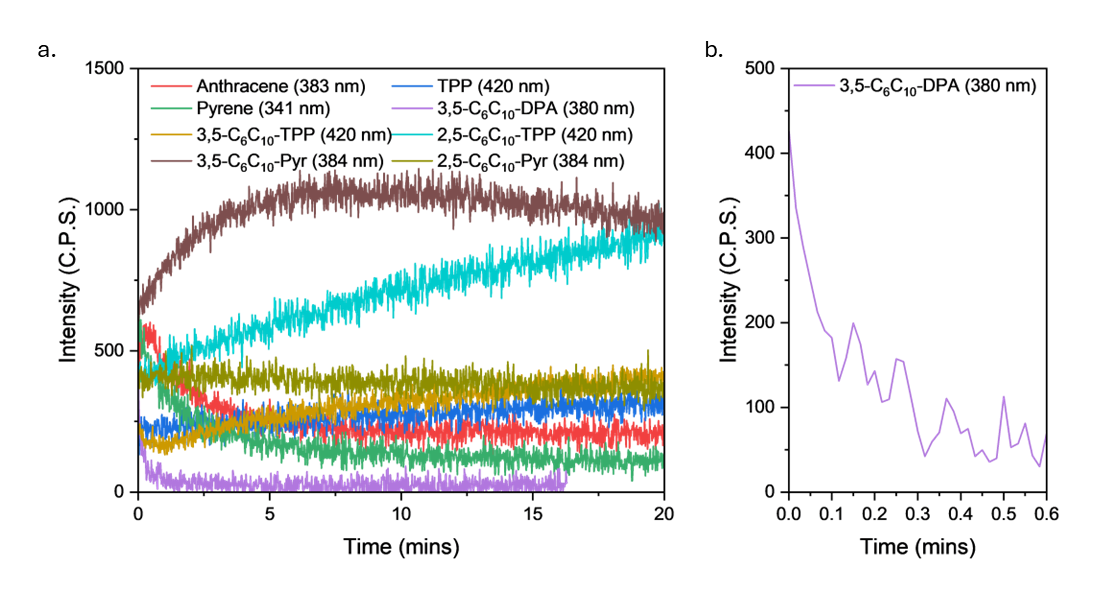


***Figure S4:*** *(a) Photostability of all compounds as thin films on quartz determined by monitoring of ^1^O_2_ photoluminescence intensity at 1270 nm during continuous irradiation at the wavelength stated in the legend. The upwards trend observed for some photosensitisers is likely due to the mobility of the compounds on the surface, with the process accelerated by an increase in surface temperature due to light irradiation. (b) Zoomed view of the photostability of* ***3,5-C_6_C_10_-DPA*** *as a thin film on quartz determined by monitoring of ^1^O_2_ photoluminescence intensity at 1270 nm during the first 0.6 mins of continuous irradiation at 380 nm.*


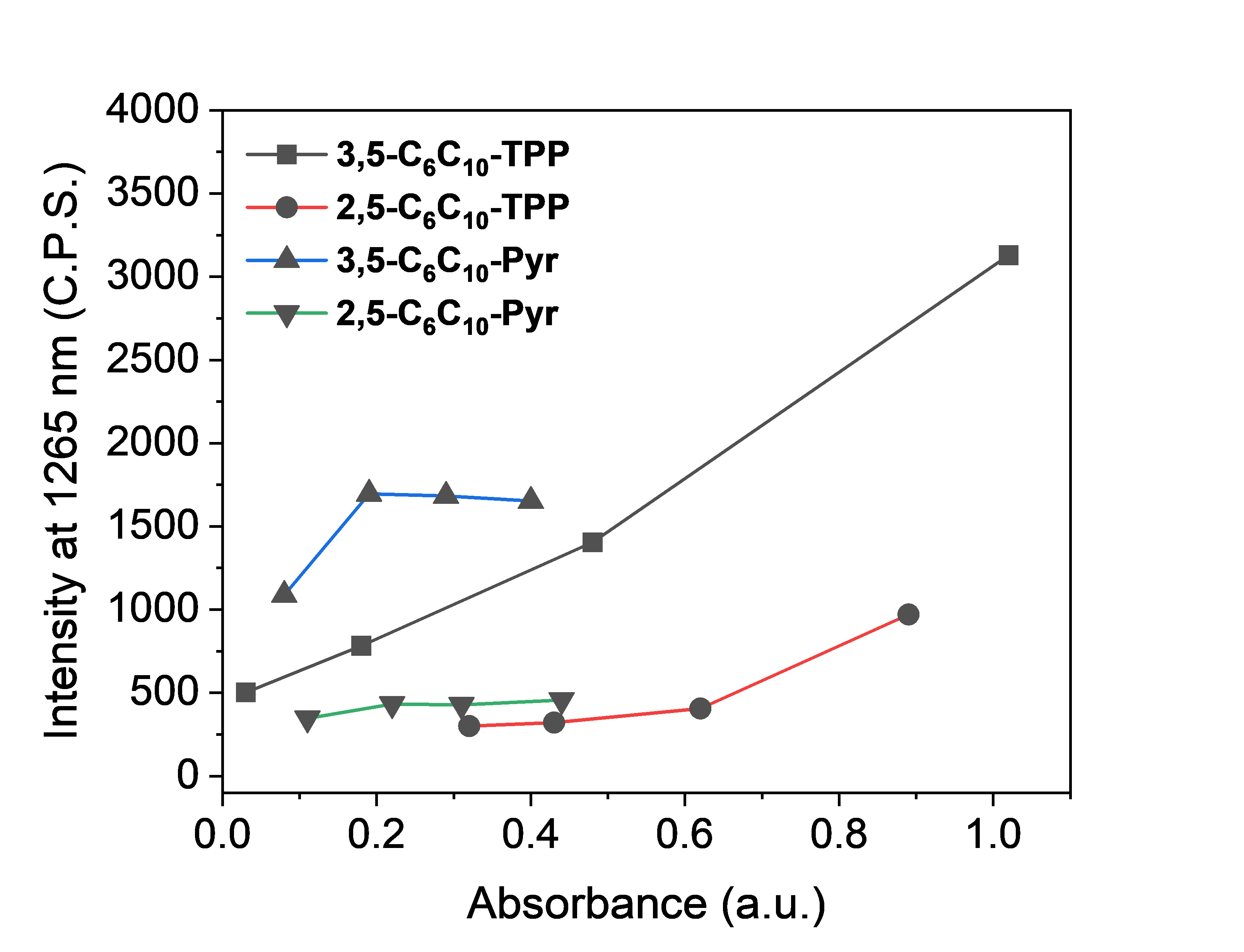


***Figure S5:*** *Correlation plot between alkyl-π liquid absorbance values and ^1^O_2_ phosphorescence intensity for spin coated thin films on quartz.*

***Table S1:*** *Summary of* ***3,5-C_6_C_1o_-TPP*** *film thickness measured by ellipsometry.*

| **3,5-TPP-C_6_C_10_** | **Thickness (nm)** | **Average thickness (nm)** |
| --- | --- | --- |
| Blank 1 | 1.673 ± 0.0121 | 1.67 ± 0.012 |
| Blank 2 | 1.688 ± 0.0122 | 1.69 ± 0.012 |
| Sample 1 - 3 mg mL^-1^ | 17.099 ± 0.0681 | 17.23 ± 0.12 |
|  | 17.358 ± 0.0616 |  |
|  | 17.166 ± 0.0554 |  |
|  | 17.398 ± 0.0554 |  |
|  | 17.162 ± 0.0647 |  |
| Sample 2 - 3 mg mL^-1^ | 16.573 ± 0.0654 | 16.43 ± 0.72 |
|  | 17.183 ± 0.0575 |  |
|  | 16.837 ± 0.0676 |  |
|  | 16.470 ± 0.0639 |  |
|  | 15.066 ± 0.0934 |  |
| Sample 1 - 6 mg mL^-1^ | 37.449 ± 0.101 | 37.58 ± 0.21 |
|  | 37.373 ± 0.101 |  |
|  | 37.625 ± 0.0982 |  |
|  | 37.961 ± 0.103 |  |
|  | 37.472 ± 0.101 |  |
| Sample 2 - 6 mg mL^-1^ | 38.109 ± 0.103 | 37.77 ± 0.31 |
|  | 37.254 ± 0.0951 |  |
|  | 37.611 ± 0.101 |  |
|  | 37.993 ± 0.105 |  |
|  | 37.899 ± 0.105 |  |
| Sample 1 - 9 mg mL^-1^ | 43.512 ± 0.349 | 54.01 ± 7.77 |
|  | 59.170 ± 0.261 |  |
|  | 63.064 ± 0.246 |  |
|  | 45.979 ± 0.348 |  |
|  | 58.346 ± 0.274 |  |
| Sample 2 - 9 mg mL^-1^ | 65.615 ± 0.197 | 65.30 ± 1.19 |
|  | 65.331 ± 0.191 |  |
|  | 65.758 ± 0.188 |  |
|  | 63.114 ± 0.202 |  |
|  | 66.706 ± 0.188 |  |
| Sample 1 - 12 mg mL^-1^ | 72.992 ± 0.185 | 75.49 ± 3.73 |
|  | 73.921 ± 0.193 |  |
|  | 82.885 ± 0.349 |  |
|  | 73.151 ± 0.272 |  |
|  | 74.533 ± 0.182 |  |
| Sample 2 - 12 mg mL^-1^ | 82.706 ± 0.202 | 81.95 ± 0.69 |
|  | 81.174 ± 0.217 |  |
|  | 81.179 ± 0.197 |  |
|  | 81.967 ± 0.198 |  |
|  | 82.756 ± 0.204 |  |
| Sample 1 - 15 mg mL^-1^ | 102.552 ± 0.285 | 101.07 ± 2.65 |
|  | 100.824 ± 0.267 |  |
|  | 96.259 ± 0.256 |  |
|  | 101.616 ± 0.286 |  |
|  | 104.126 ± 0.285 |  |
| Sample 2 - 15 mg mL^-1^ | 102.167 ± 0.326 | 103.53 ± 3.5 |
|  | 102.413 ± 0.273 |  |
|  | 102.180 ± 0.27 |  |
|  | 110.369 ± 0.253 |  |
|  | 100.462 ± 0.416 |  |
| Sample 1 - 18 mg mL^-1^ | 117.799 ± 0.254 | 118.36 ± 1.86 |
|  | 121.142 ± 0.24 |  |
|  | 115.660 ± 0.262 |  |
|  | 117.632 ± 0.248 |  |
|  | 119.570 ± 0.255 |  |
| Sample 2 – 18 mg mL^-1^ | 119.890 ± 0.263 | 118.73 ± 1.24 |
|  | 119.265 ± 0.259 |  |
|  | 117.549 ± 0.262 |  |
|  | 119.987 ± 0.261 |  |
|  | 116.968 ± 0.271 |  |


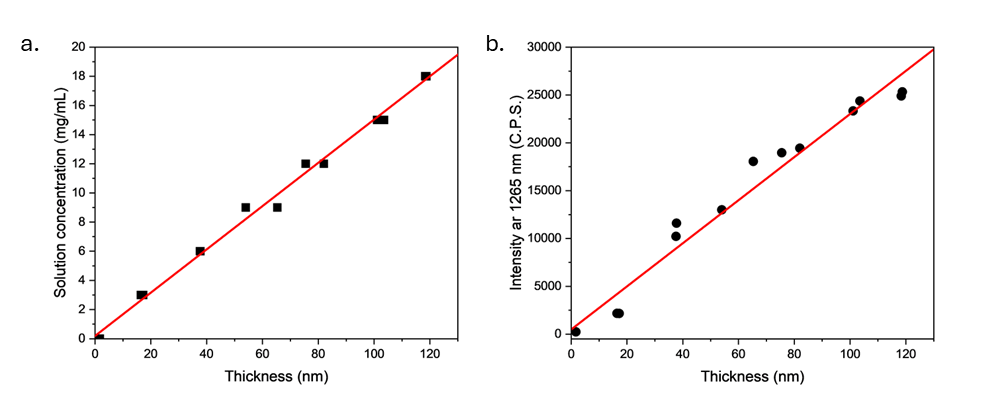


***Figure S6:*** *Correlation plot between film thickness measurement by ellipsometry and the concentration (mg mL^-1^) of the solution of* ***3,5-C_6_C_1o_-TPP*** *used for spin coating.*


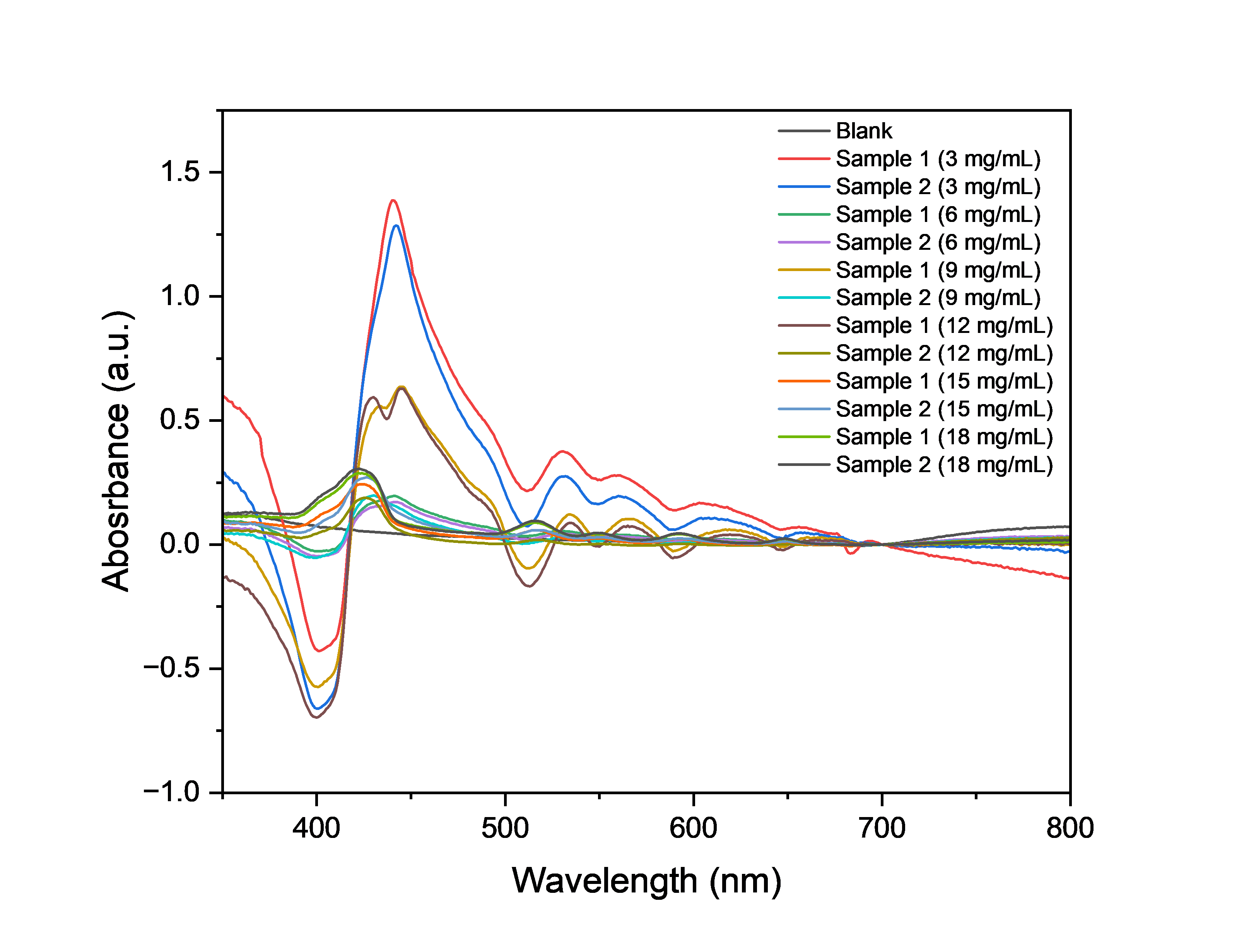


***Figure S7:*** *UV-vis spectra of* ***3,5-C_6_C_1o_-TPP*** *as thin films on silicon wafers produced from spin coated 0 - 18 mg mL^-1^ chloroform solutions, recorded using an integrated sphere.*

*
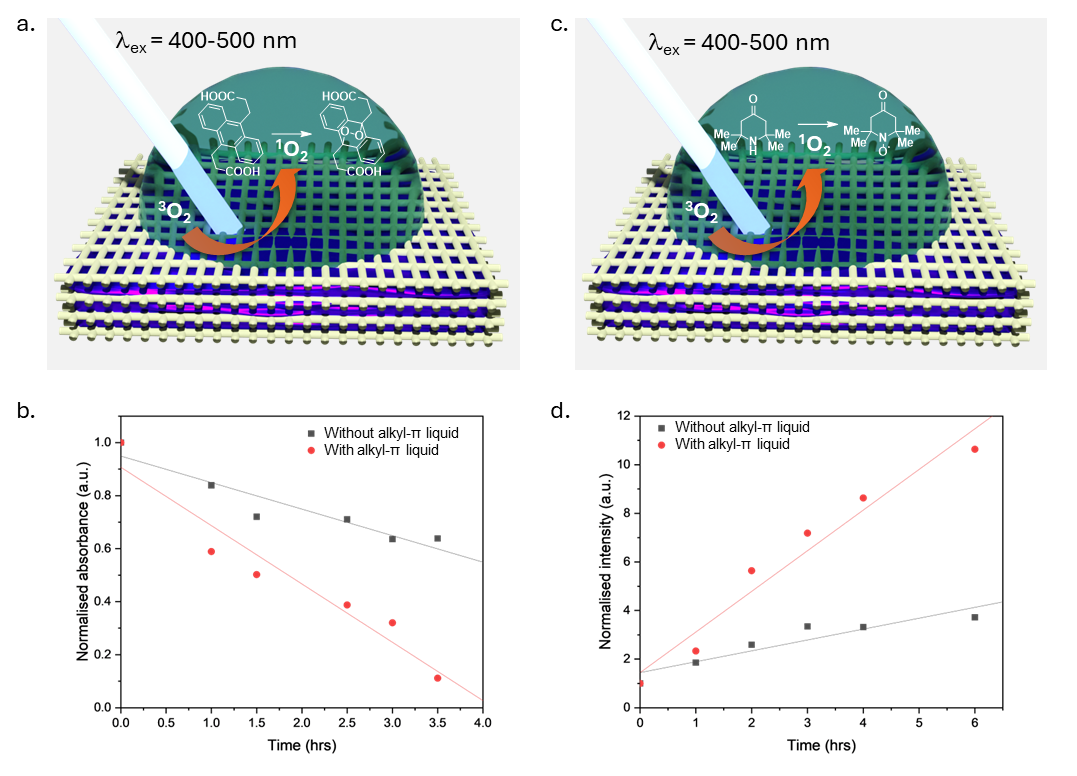
*

***Figure S8:*** *Rate of ^1^O_2_ production for* ***3,5-C_6_C_1o_-TPP*** *impregnated membranes. (a) Schematic of the experimental setup for ^1^O_2_ detection using an anthracene probe and UV-vis spectroscopy. (b) Correlation plot between time and normalised absorbance with linear fit lines for membrane samples with and without* ***3,5-C_6_C_1o_-TPP*** *measured by monitoring the degradation of an anthracene probe by UV-vis spectroscopy. (c) Schematic of the experimental setup for ^1^O_2_ detection using TEMPD spin trap and EPR spectroscopy. (d) Correlation plot between time and normalised intensity with linear fit lines for membrane samples with and without* ***3,5-C_6_C_1o_-TPP*** *measured by monitoring the formation of a stable radical of TEMPD by EPR spectroscopy.*
